# Contribution of allergy in the acquisition of uncontrolled severe asthma

**DOI:** 10.3389/fmed.2022.1009324

**Published:** 2022-09-21

**Authors:** María Isabel Delgado Dolset, David Obeso, Juan Rodriguez-Coira, Alma Villaseñor, Heleia González Cuervo, Ana Arjona, Coral Barbas, Domingo Barber, Teresa Carrillo, María M. Escribese

**Affiliations:** ^1^Institute for Applied Molecular Medicine Nemesio Díez, School of Medicine, Universidad San Pablo-CEU, CEU Universities, Urbanización Montepríncipe, Boadilla del Monte, Spain; ^2^Centre of Metabolomics and Bioanalysis (CEMBIO), School of Pharmacy, Universidad San Pablo-CEU, CEU Universities, Urbanización Montepríncipe, Boadilla del Monte, Spain; ^3^Allergy Service, Hospital Universitario de Gran Canaria Doctor Negrin, Las Palmas de Gran Canaria, Spain; ^4^Department of Medical and Surgical Sciences, School of Health Sciences, Universidad de Las Palmas de Gran Canaria, Las Palmas de Gran Canaria, Spain; ^5^Department of Basic Medical Sciences, School of Medicine, Universidad San Pablo-CEU, CEU Universities, Urbanización Montepríncipe, Boadilla del Monte, Spain

**Keywords:** asthma, metabolomics, allergy, lysophospholipids, bile acids (BAs), HDM-allergy

## Abstract

Asthma is a multifactorial, heterogeneous disease that has a challenging management. It can be divided in non-allergic and allergic (usually associated with house dust mites (HDM) sensitization). There are several treatments options for asthma (corticosteroids, bronchodilators, antileukotrienes, anticholinergics,…); however, there is a subset of patients that do not respond to any of the treatments, who can display either a T2 or a non-T2 phenotype. A deeper understanding of the differential mechanisms underlying each phenotype will help to decipher the contribution of allergy to the acquisition of this uncontrolled severe phenotype. Here, we aim to elucidate the biological pathways associated to allergy in the uncontrolled severe asthmatic phenotype. To do so, twenty-three severe uncontrolled asthmatic patients both with and without HDM-allergy were recruited from Hospital Universitario de Gran Canaria Dr. Negrin. A metabolomic fingerprint was obtained through liquid chromatography coupled to mass spectrometry, and identified metabolites were associated with their pathways. 9/23 patients had uncontrolled HDM-allergic asthma (UCA), whereas 14 had uncontrolled, non-allergic asthma (UCNA). 7/14 (50%) of the UCNA patients had Aspirin Exacerbated Respiratory Disease. There were no significant differences regarding gender or body mass index; but there were significant differences in age and onset age, which were higher in UCNA patients; and in total IgE, which was higher in UCA. The metabolic fingerprint revealed that 103 features were significantly different between UCNA and UCA (*p* < 0.05), with 97 being increased in UCA and 6 being decreased. We identified lysophosphocholines (LPC) 18:2, 18:3 and 20:4 (increased in UCA patients); and deoxycholic acid and palmitoleoylcarnitine (decreased in UCA). These metabolites were related with a higher activation of phospholipase A2 (PLA2) and other phospholipid metabolism pathways. Our results show that allergy induces the activation of specific inflammatory pathways, such as the PLA2 pathway, which supports its role in the development of an uncontrolled asthma phenotype. There are also clinical differences, such as higher levels of IgE and earlier onset ages for the allergic asthmatic group, as expected. These results provide evidences to better understand the contribution of allergy to the establishment of a severe uncontrolled phenotype.

## Introduction

Asthma is a heterogenous, multifactorial respiratory disease that is characterized by wheezing, shortness of breath, chest tightness and coughing (1). It affects around 1–18% of the population worldwide, significantly reducing their quality of life (2).

Asthma management is a challenge due to its complexity. In fact, there are multiple phenotypes, which are defined according to the clinical manifestation of the disease, as well as endotypes defined according to the underlying mechanisms. Thus, efforts to define homogenous phenotypes of asthma based on their underlying characteristics, have been pushed in the last years (3–5).

Allergic asthma has been deeply studied over the years. Around 80% of childhood-onset asthma and more than 50% of asthmatic adult patients suffer from allergic asthma (6). It has an early onset, usually in childhood, that commonly persists throughout life (3, 7), and is characterized by a type 2 inflammatory profile, dominated by IL-4, IL-5, IL-9 and IL-13, Th2 cells, eosinophils, and IgE producing plasma cells (8). On the other hand, non-allergic or intrinsic asthma is poorly understood (9). It usually appears in adulthood, normally around 30–40 years old (10). Inflammatory profiles vary among patients, which can be type 2-high or low; and multiple effector cells might be involved, including eosinophils, neutrophils, both, or none (paucigranulocitic asthma) (4, 11). Moreover, it is usually associated with aspirin-exacerbated respiratory disease (AERD), a non-allergic intolerance to non-steroidal anti-inflammatory drugs (NSAIDs) related to cyclooxygenase (COX)-1. Patients with AERD experiment worsened asthma symptoms while taking this kind of medications, and they usually present nasal polyps (12, 13).

Treatment for asthmatic patients is complex and is prescribed in a step by step approach regarding GINA guidelines (1). Mild allergic asthmatic patients' symptoms control involves either topical or inhaled corticosteroids, usually in combination with short- and long-acting bronchodilators, antileukotrienes, anticholinergics, among other pharmacological drugs, and/or allergen-specific immunotherapy (AIT); while for the more severe ones, systemic corticosteroids and add-on biological therapy targeting type 2 inflammation mediators are also needed. Currently, anti-IgE (omalizumab), anti-IL-5 (mepolizumab, reslizumab), anti-IL-5Rα (benralizumab) and anti-IL-4Rα (dupilumab) are approved for asthmatic patients (14), and others, such as anti-TSLP (tezepelumab) are currently under research. Still, there is a subset of asthmatic patients that do not respond to any of these treatments and remain uncontrolled, suffering several exacerbations and hospitalizations every year, which leads to a very low quality of life. In fact, this subset of patients involves allergic and non-allergic patients.

On the other hand, type 2-low non-allergic asthmatic patients do not have any medication that specifically targets their underlying characteristics. Although macrolides have used to treat these patients, they have shown limited success; and a continued use is frowned upon due to antibiotic resistance dangers (15).

Overall, uncontrolled asthma remains a concern, as these patients usually present more comorbidities, a lower quality of life and higher mortality rates (16). Additionally, evidence regarding the role of allergy in the evolution of asthmatic severity is still lacking.

Allergic asthma usually presents sensitization to house dust mites (HDM). There are reports that up to 85% of asthmatic patients are sensitized to HDM (17). It has been recently shown that sensitization to HDM is associated with asthma severity and a lack of disease control in children (18). HDM are arachnids that live in tropical areas with high humidity and temperate climates (19, 20); but are present worldwide and widely populate clothes and bed linens (21). Their association with asthma is hypothesized to be related to the physicochemical properties of HDM allergens (such as Der p 1, Der p 2, Der p 21), which include proteases and immune system mimickers that can activate TLR4 responses (22). Thus, these proteins might be able to, first, damage the epithelium, and then, activate the immune system, eliciting an immune response, especially in children with an immature epithelial barrier. In fact, respiratory infections in childhood are associated with a higher risk of developing asthma (7). Moreover, it has been recently described that Der p 1 elicits a differential metabolic response in an *in vitro* model of lung epithelium between underdeveloped (2 days of culture) and mature (7 days of culture) tissue (23).

However, despite the fact that allergic asthma is the most common phenotype of asthma, and that sensitization to HDM is the most common among these patients, there are asthmatic patients that do not have concomitant allergy, even in areas where HDM exposure is high, such as tropical climates (24) or high-humidity areas such as islands. One of these regions is the Canary Islands, in Spain, where the HDM exposome has been characterized in depth (19, 20, 25, 26). The reason why some patients do not get sensitized to HDM, but do develop severe uncontrolled asthma without allergy, is unknown; although the fact that lots of these patients have a family history of asthma might be related. Also unknown are the implications of allergy in the development and evolution of asthma. Thus, we aim to decipher the role of allergic inflammation in the pathogenesis of uncontrolled asthma.

## Materials and methods

### Patients

The study was approved by the Ethics Committee of the Hospital on 4^th^/February/2016 (code: 160009). Twenty-three severe uncontrolled asthmatic patients with and without allergy were recruited in the Hospital Universitario de Gran Canaria doctor Negrin (Las Palmas de Gran Canaria, Spain). Allergic status was assessed by skin-prick test to a set of HDM allergens (*Dermatophagoides pteronyssinus, Dermatophagoides farinae, Blomia tropicalis, Acarus siro, Lepidoglypus destructor* and *Tyrophagus putrescentiae*) and the analysis of clinical history; and patients were classified as allergic, if they were sensitized to HDM (and, possibly, to other allergens) and as non-allergic if they had no known sensitizers. Severity was defined according to the GINA guidelines; patients included were at least on step 4–5 of medication and did not respond to the treatments approved at the date of inclusion (including high doses of inhaled and systemic corticosteroids and/or anti-IgE). They also had around 5 exacerbations per year.

Differences in age, onset age, BMI and total IgE in patients were analyzed using Mann-Withney U test, as the allergic group had <10 patients, while gender, number of smokers and number of patients with AERD were analyzed by Fisher's Exact, using GraphPad Prism v9.3.1 for Windows (GraphPad Software, San Diego, California USA, www.graphpad.com).

### Metabolomic analysis

The metabolomic analysis has already been thoroughly described elsewhere (27, 28). Briefly, serum samples were measured in batches using an Agilent High Performance Liquid Chromatography system (Agilent 1200 series) coupled with a quadrupole- time of flight (QTOF) analyser system (Q-ToF MS 6520) (Agilent Technologies, Waldbronn, Germany), in positive and negative ionization modes (ESI + and ESI -, respectively). A quality control sample (QC) prepared by mixing equal volumes of a set of samples was measured throughout the analytical run. The HPLC system was equipped with a degasser, two binary pumps, and a thermostated autosampler. For the analysis, 10 μL of sample were injected into a Discovery HS C18 (2.1 × 150 mm, 3.0μm; Supelco, Sigma Aldrich, Germany), maintained at 40°C. The flow rate was set at 0.6 mL/min. The elution gradient involved a mobile phase consisting of: (A) 0.1% v/v formic acid (FA) in water and (B) 0.1% v/v FA in acetonitrile. The initial conditions were set at 25% phase B, which increased linearly to 95% phase B in 35 min, and then returned to the initial conditions in 1 min, which were held for 9 min for column reconditioning. Samples were analyzed in both ESI+ and ESI− modes. The capillary voltage was set at 3500 for ESI+ and 4000V for ESI−. The drying gas flow rate was 10.5 L/min at 330°C and gas nebulizer at 52 psi; fragmentor voltage was 175 V; skimmer and octopole radio frequency voltages were set to 65 and 750 V, respectively. A full scan from 100 to 1200 *m/z* for both modes was performed. MS spectra were collected in the centroid mode at a scan rate of 1.2 Hz. Automatic MS recalibration during batch analysis was carried out introducing a reference standard into the source via a reference sprayer valve. Reference masses for ESI+ were purine (*m/z* = 121.0508) and HP-0921 (*m/z* = 922.0097), whereas for ESI− TFA were NH4 (*m/z* = 119.0363) and HP-0921 (*m/z* = 966.0007).

Multivariate analysis was performed using SIMCA v.16.0 (Sartorius Stedim Data Analytics). A Principal Component Analysis (PCA) model was used to evaluate data quality and find patterns in samples. Likewise, Partial Least Square Discriminant Analysis (PLS-DA) and Orthogonal Projections to Latent Structures Discriminant Analysis (OPLS-DA) supervised models were used to classify the samples and to evaluate differences between groups. Models were evaluated using R^2^ and Q^2^ parameters which are the classification and prediction capacity, respectively.

Significant features were selected after pair-wise comparisons using two-tailed Mann-Whitney U test with an FDR correction using an in-house script for Matlab R2015a software (Mathworks, Natick, Massachusetts, USA); and used to build a Hierarchical Clustering Heatmap (HCA), with MetaboAnalyst 5.0 online tool (https://www.metaboanalyst.ca).

Metabolite annotation was performed by comparison with online platform CEU Mass Mediator 3.0 (29–32) for data bases, and confirmed through tandem mass spectrometry (MS/MS). LC-MS/MS was performed in a similar LC-Q-ToF-MS instrument than the original experiment (Agilent series 1290 HPLC and series 6550 Q-ToF, respectively); and the method for the new equipment, which has been previously described (33, 34), was adapted to be as close as possible to the original method used. In this analysis, the HPLC system was equipped with a degasser, two binary pumps, and a thermostated autosampler. Briefly, 2 μL of sample were injected into a Zorbax C18-Extend USH BD06804 column (2.1 × 150 mm, 5.0 μm; Agilent Technologies), maintained at 60 °C. The flow rate was set at 0.6 mL/min. The elution gradient involved a mobile phase with the same components than those of the previous analysis, and the initial conditions were set at 5% phase B, which increased linearly to 80% phase B in 7 min, and then to 100% phase B in 4.5 min. Then it returned to the initial conditions in 0.5 min, which were held for 3 min. The capillary voltage was set at 3000 for ESI+ and 4000V for ESI−. The drying gas flow rate was 12 L/min at 250°C. and gas nebulizer, fragmentor voltage, skimmer and octupole radio frequency were set as in the LC-MS analysis. MS/MS spectra were collected in the centroid mode; ions were targeted using the narrow m/z window (1.3 Da) and 20 eV of energy for fragmentation on the quadrupole. To obtain the newer retention time (RT) for this shorter method, samples were first run for LC-MS; then, *m/z* were matched with their newer RT. Some of the peaks could not be matched with the new RTs. Finally, comparison of the structure proposed against the obtained fragments led to the confirmation of the identity.

An enrichment analysis was performed using MetaboAnalyst 5.0 online tool (https://www.metaboanalyst.ca). Moreover, pathways of the significant identified compounds were obtained using IMPaLA (v 12.0) online tool (http://impala.molgen.mpg.de/).

## Results

### Patients

A total of 23 patients, 9 allergic to HDM and 14 non-allergic, were recruited for metabolomic analysis. Clinical characteristics of the groups can be found on Table 1.

**Table 1:** Clinical characteristics of the recruited patients.

	**UCNA**	**UCA**
*n*	14	9
Age	62.1 ± 2.8	47 ± 4.7^**^
Onset age	29.4 ± 2.9	9.6 ± 2.1^****^
Sex (F/M)	13/1	6 / 3
BMI	29.7 ± 1.4	27.3 ± 1.4
Current smokers	0	0
Total IgE	257.9 ± 138.2	683.2 ± 222.2^**^
AERD (%)	7 (50%)	0

BMI, body mass index; AERD, Aspirin Exacerbated Respiratory Disease.

^*^p-value < 0.05;

** *p*-value < 0.01;

^***^p-value < 0.001;

**** *p*-value < 0.0001.

Non-allergic uncontrolled asthmatic patients (UCNA) were older and had a later onset of asthma than allergic uncontrolled asthmatic patients (UCA) (62.1 ± 2.8 *vs*. 47 ± 4.7, *p* < 0.01, and 29.4 ± 2.9 *vs*. 9.6 ± 2.1, *p* < 0.0001, respectively). UCNA also had lower levels of total IgE (257.9 ± 138.2 *vs*. 683.2 ± 222.2, *p* < 0.01), as expected. None of the patients were active smokers.

Moreover, 7 (50%) of the UCNA patients suffered AERD, 1 (7%) reported nasal polyps without NSAIDs hypersensitivity besides asthma, and 1 (7%) suffered from NSAIDs hypersensitivity without nasal polyps. On the other hand, from UCA patients, only 1 (11%) had NSAIDs hypersensitivity, and none had nasal polyps.

Overall, our findings, as expected, demonstrate significant differences in clinical and demographic features between both groups.

### Metabolomic analysis reveals distinct phenotypes for allergic and non-allergic uncontrolled asthma

**Figure 1:**
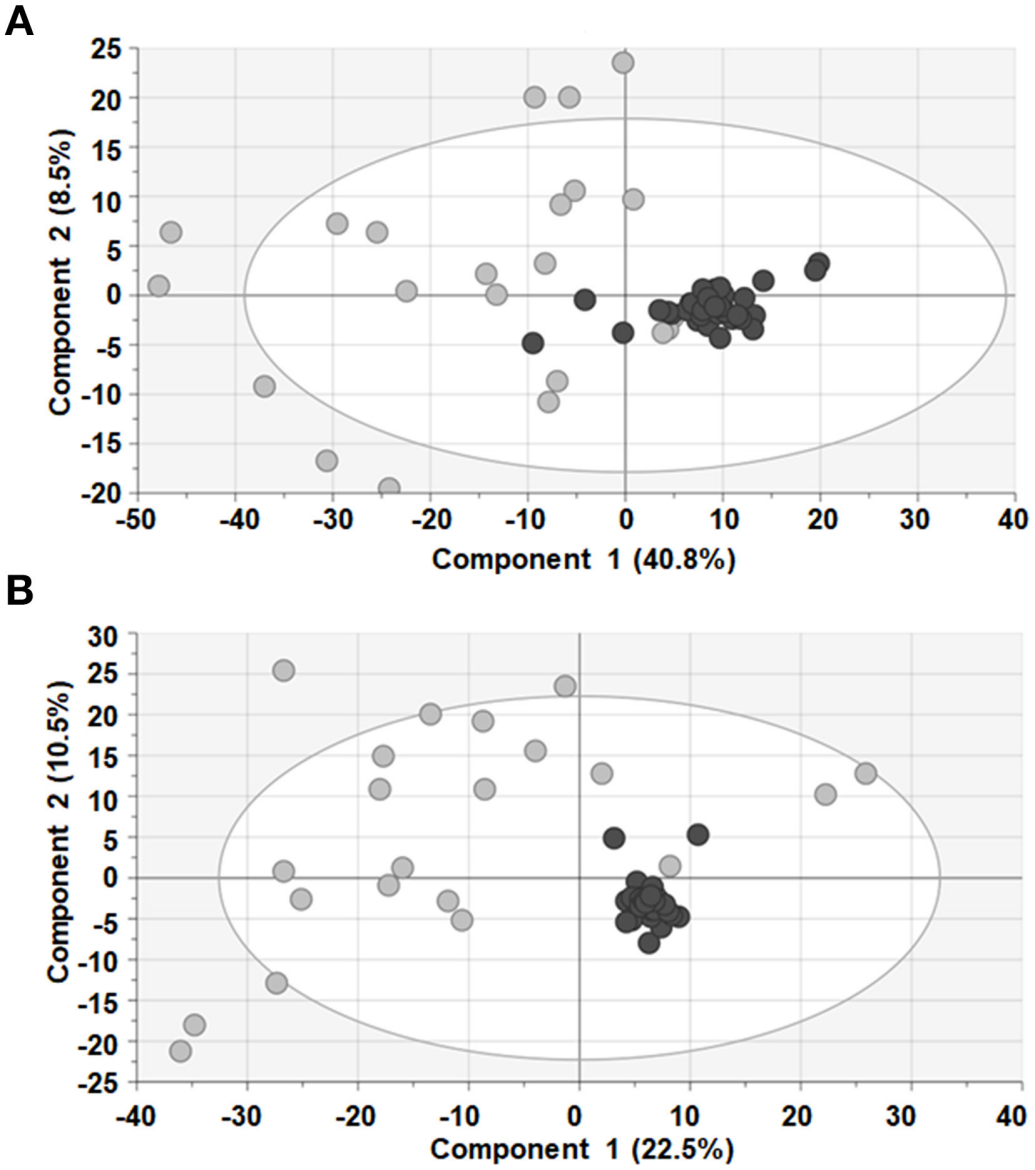
PCA models showed that QC samples (black dots) clustered together in both ESI+ **(A)** and ESI− **(B)**, ensuring quality of the data. Sample of patients (gray dots) are also shown.

**Figure 2:**
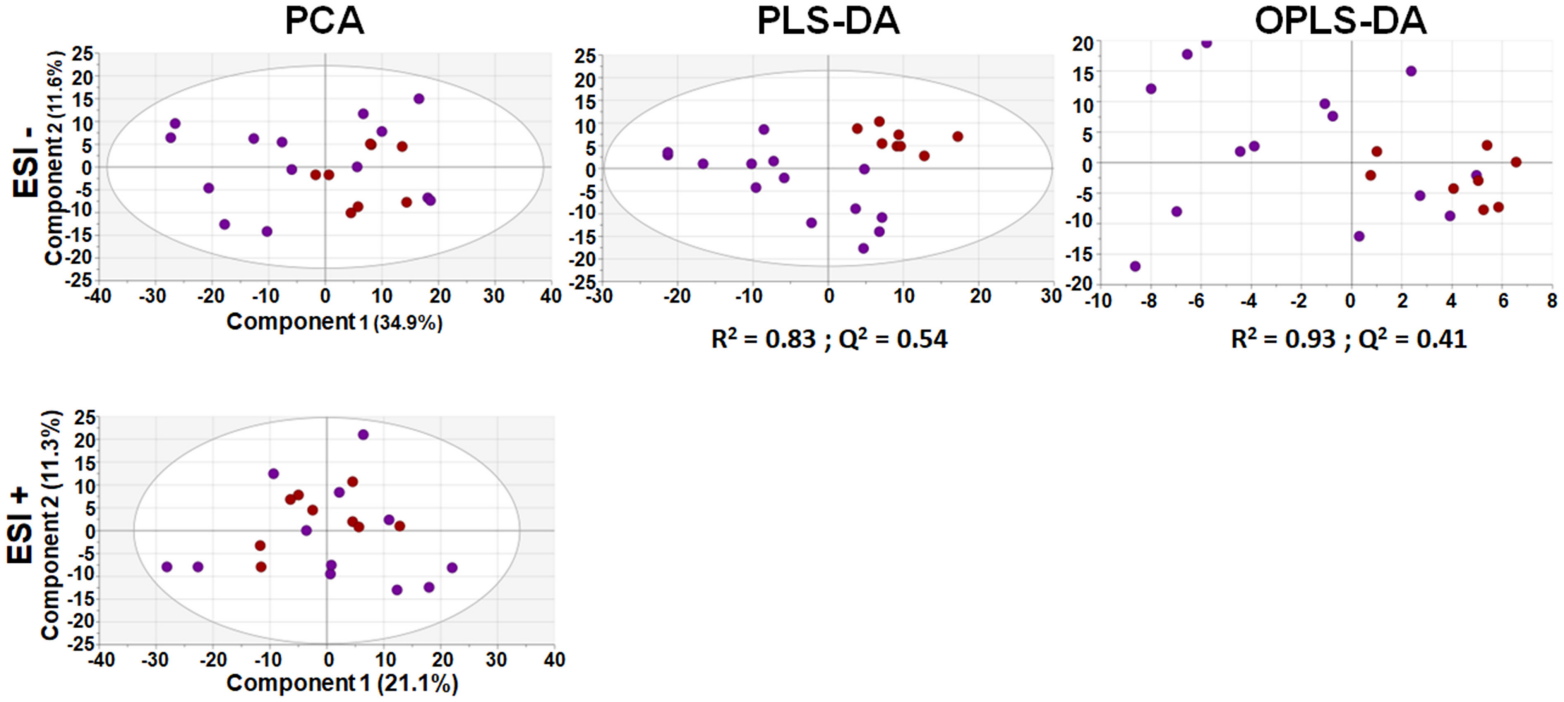
Multivariant analysis of metabolomic data from serum samples. An unsupervised PCA model (let) of UCA and UCNA patients was built using 593 features for ESI – (above) and 734 for ESI + (below). Then, supervised PLS-DA (center) and OPLS-DA (right) models were built; but only models for the ESI– mode were found. All data were UV scaled. UCA, red dots; UCNA, purple dots. *R*^2^ is the capability of the model to classify the samples; *Q*^2^ is the capability of the model to predict the class of a new sample.

After appropriate data processing, a total number of 1,327 features (593 for ESI– and 734 for ESI+) were obtained for the metabolomic profiling of serum. Quality of the data was assessed by grouping of QC samples on a non-supervised PCA model (Figure 1) proving that the variability of the samples was biological and not due to the technique. Then, both groups were compared using a multivariant analysis (Figure 2). We found that UCA patients tended to cluster in the ESI- plot (Figure 2, left) even if the PCA models did not completely separate both groups. As for the PLS-DA and OPLS-DA supervised models, only the ones obtained with the ESI- mode data were valid (upper part of Figure 2); and they had R^2^ and Q^2^ parameters of 0.85 and 0.55, for the PLS-DA mode (Figure 2, center), and 0.91 and 0.4, on the OPLS-DA model, where 100% of UCA samples and 64% of UCNA were correctly classified (Figure 2, right).

**Figure 3:**
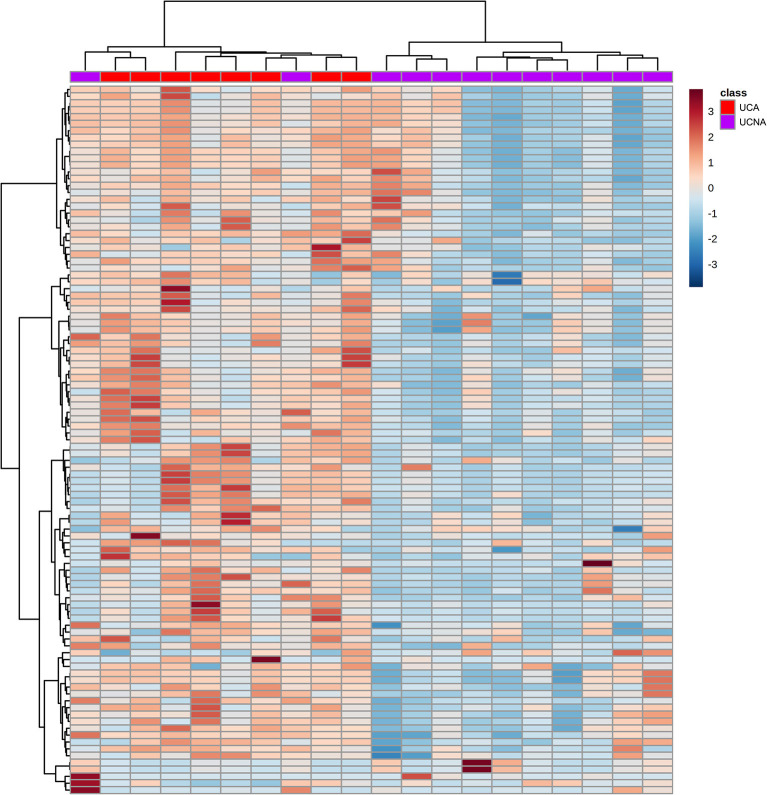
Hierarchical clustering analysis heatmap of the UCA (red) and UCNA (purple) patients (in columns) built using the 103 significantly different signals between the groups (in columns). Samples and metabolites were clustered according to their similarity. Red and blue cells represent an increase or decrease in the abundance of a given metabolite.

Afterwards, we performed an univariant analysis with Mann Whitney-U (as the allergic group had <10 patients) and obtained a total of 83 (for ESI–) and 20 (for ESI+) significantly different signals. Most of them (97) were increased in UCA patients compared to UCNA patients. These 103 features were used to build HCA heatmap (Figure 3). As shown, this model was able to correctly classify most of the patients (21 out of 23), grouping all UCA + 2 UCNA patients together (on the left) and the rest of the UCNA patients on the other branch.

### Lipid metabolism, specifically for steroids and phosphocholines, lead the differences among groups

**Figure 4:**
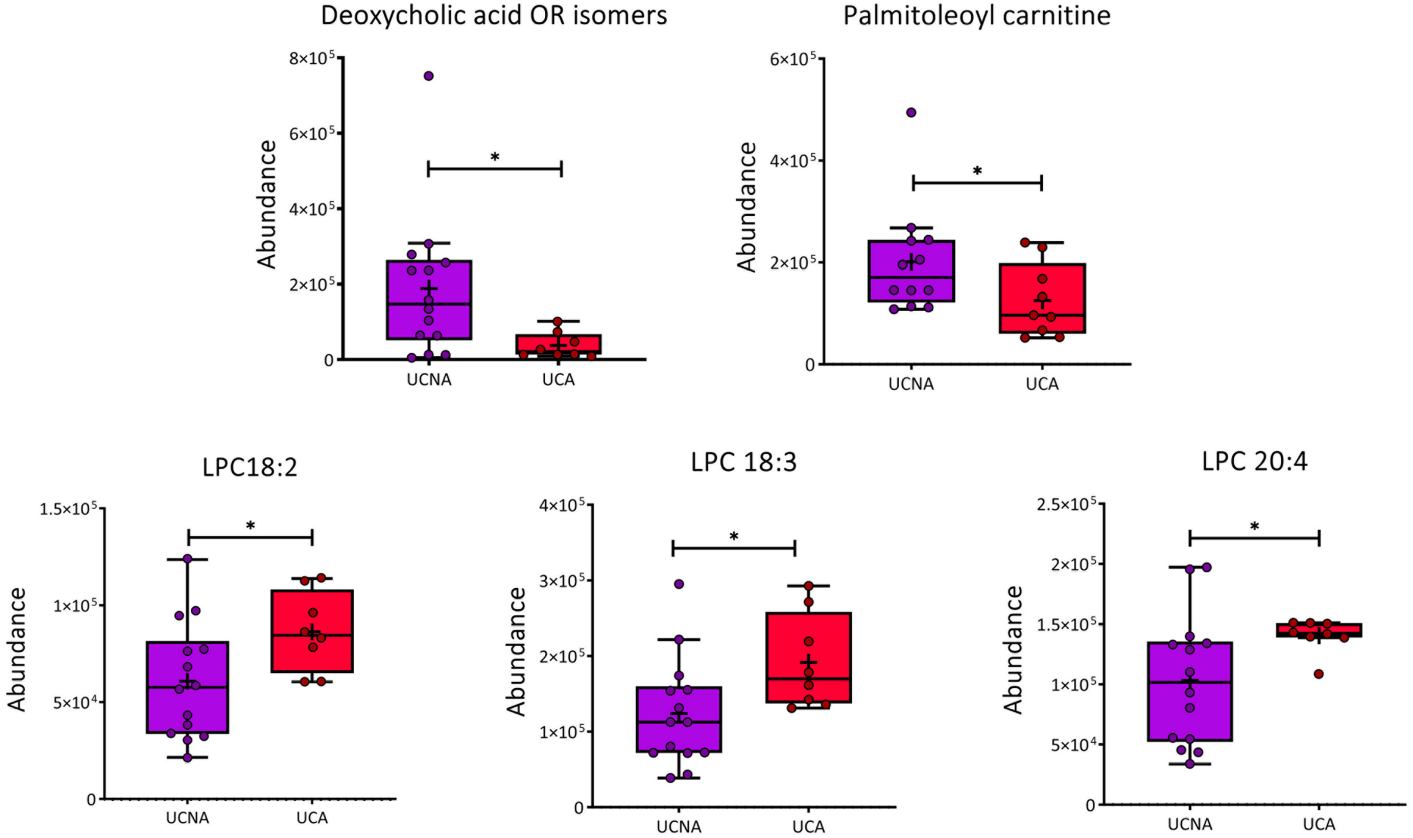
Trajectories of relevant identified metabolites in box and whiskers plots between UCA (red) and UCNA (purple). Mean is represented by “+” inside the boxes, and individual data points are shown in dots. Mann–Witney *U* test was used to calculate significant differences. *p < 0.05.

We wanted to identify which were the specific features responsible for the separation of these groups. Thus, we performed a tandem MS/MS analysis and compared the obtained fragmented features with fragmentation spectra when available. In addition, manual identification was performed based on previous publications (35–37). From the 103 features, we were able to identify 14, with 13 of them having a unique *m/z*@RT (Table 2). These included steroids (hormones, bile acids, vitamins), lysophosphocholines, and a carnitine, which were all increased in UCA compared to UCNA, with the exception of deoxycholic acid and palmitoleoylcarnitine, which were decreased in UCA. Trajectories of some relevant metabolites can be found in Figure 4.

**Table 2:** Physicochemical characteristics of significant annotated signals in metabolomics through LC-MS/MS between UCNA and UCA groups.

**No**.	**Technique**	**Biological category**	**Compound**	**m/z**	**Mass (Da)**	**RT (min)**	**Formula**	**Error (ppm)**	**Adduct**	**CV in QCs (%)**	**% Change (UCA)**	***p*-value**	***p*-value (BH)**
1	LC-MS-	Vitamins	1α,25-dihydroxy-2α-(3-hydroxypropoxy)vitamin D3 OR isomers	489.3552	490.363	30.47	C30H50O5	−5.7	M-H	6.7	37.3	0.0105	0.1298
2	LC-MS-	Steroids	5α-Dihydrotestosterone sulfate OR isomers	369.1738	370.1816	4.59	C19H30O5S	0.5	M-H	6.3	74.3	0.0070	0.1021
3	LC-MS-	Steroids	5α-Dihydrotestosterone sulfate OR isomers	369.1736	370.1814	8.52	C19H30O5S	0	M-H	5.7	77	0.0070	0.1021
4	LC-MS-	Steroids	5α-Dihydrotestosterone sulfate OR isomers	369.1738	370.1816	10.63	C19H30O5S	0.5	M-H	7.4	78.7	0.0007	0.0915
5	LC-MS-	Steroids	17α,20α-Dihydroxycholesterol OR isomers	463.3426	418.3449	29.92	C27H46O3	0.5	M+FA-H	11.3	36	0.0154	0.1629
6	LC-MS-	Steroids	Androsterone 3-glucuronide OR isomers	465.2496	466.2574	7.81	C25H38O8	1.5	M-H	6.2	66.1	0.0030	0.0915
7	LC-MS-	Steroids	Androsterone 3-glucuronide OR isomers	465.2463	466.2541	8.1	C25H38O8	−5.6	M-H	6.2	52.6	0.0127	0.1453
8	LC-MS-	Steroids	Androsterone 3-glucuronide OR isomers	465.2474	466.2552	9.61	C25H38O8	−3.2	M-H	9.8	27	0.0154	0.1629
9	LC-MS-	Bile acids	Deoxycholic acid OR isomers	437.2901	392.2924	14.73	C24H40O4	−0.5	M+FA-H	5.4	−385.8	0.0222	0.2053
10	LC-MS-	Phospholipids	Phosphocholine (18:2/0:0)	504.3085	519.332	16.97	C26H50NO7P	−0.9	M-CH3	9.6	29.5	0.0374	0.2862
11	LC-MS-	Phospholipids	Phosphocholine (18:3/0:0)	562.3137	517.316	15.63	C26H48NO7P	−1.5	M+FA-H	12.7	35.3	0.0374	0.2862
12	LC-MS-	Phospholipids	Phosphocholine (20:4/0:0)	656.3182	543.3332	17.12	C28H50NO7P	1.2	M+TFA-H	27.4	26.6	0.0265	0.2272
13	LC-MS-	Phospholipids	Phosphocholine (0:0/20:4)	614.3483	569.3506	18.59	C30H52NO7P	4.4	M+FA-H	20.9	35.7	0.0265	0.2272
14	LC-MS+	Carnitines	Palmitoleoyl carnitine	398.3269	397.3191	15.95	C23H43NO4	−0.3	M+H	11.3	−60.5	0.0428	0.9027

FA, Formic Acid; TFA, Trifluoroacetic acid; CV, Coefficient of variation; QC, Quality control. The CV in the injections of the QC sample assures that the metabolites were measured throughout the experiment in a constant and reproducible manner. In this sense, if the variations in QCs are lower than 30%, it means that the variations found in a particular metabolite are due to the true increase or decrease between the comparison groups. Thus, the % of CV in QCs should be always less than the % change between groups for each metabolite, which assesses that the results of this experiment are reliable.

**Figure 5:**
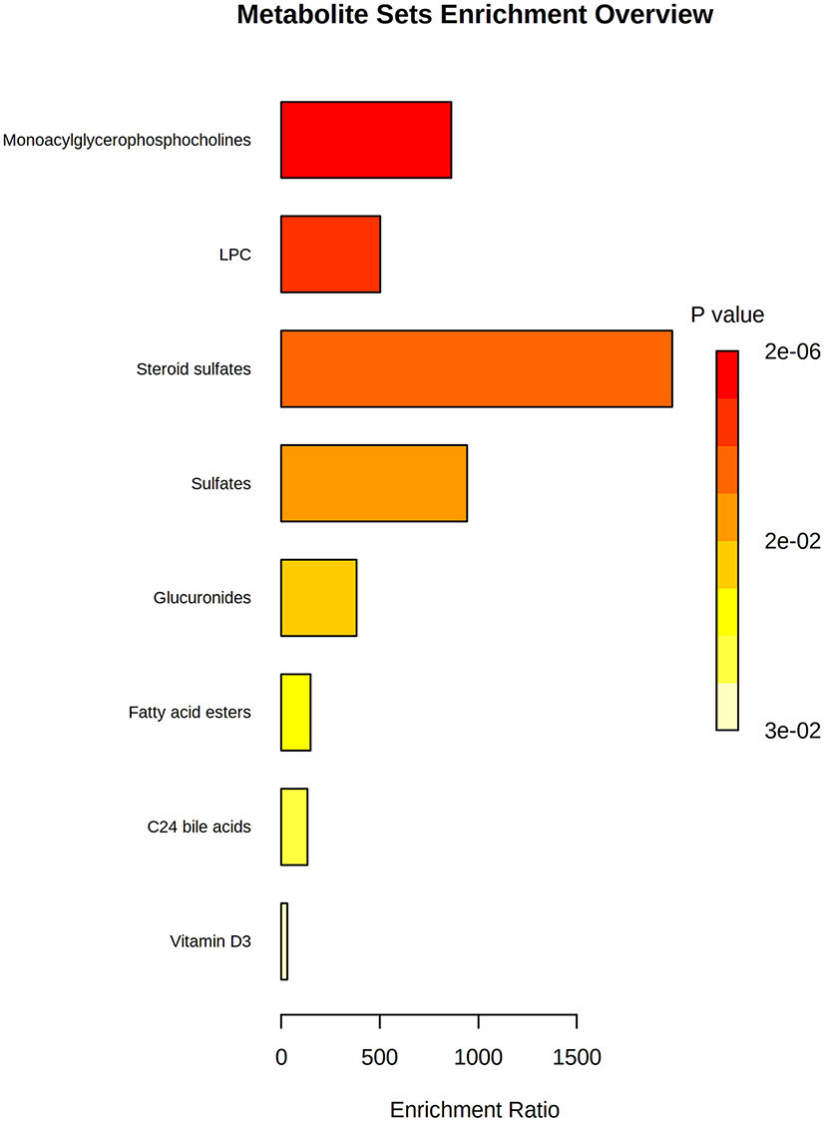
Enrichment analysis of the most changed biological categories for the altered compounds. *P-*value is shown by a yellow-red color scale; and the relevance of the change is shown by an enrichment ratio.

Finally, we performed an enrichment analysis to obtain the chemical subclasses that were changed in our samples (Figure 5); where the more changed classes were lysophosphocholines (LPC) and steroids.

Moreover, we tried to relate these specific compounds to the pathways in which they are involved using IMPALA (Table 3). We found 155 possible related pathways, 44 of them being significantly different, including the phospholipase A2 (PLA2) pathway, steroid hormone biosynthesis, phosphatidyl choline catabolism and acyl chain remodeling, or lipids metabolism, among others.

**Table 3:** Significantly enriched metabolic pathways in UCA patients compared to UCNA (*p* < 0.05).

**Pathway name**	***p*-value**	***p*-value (BH)**	**Number of genes identified in pathway**	**Pathway source**
Osteoblast signaling	0.00383	1	1	Wikipathways
Retinoic acid receptors-mediated signaling	0.00383	1	1	PID
Signaling events mediated by the hedgehog family	0.00383	1	1	PID
Rheumatoid arthritis - Homo sapiens (human)	0.00574	1	1	KEGG
STING pathway in kawasaki-like disease and COVID-19	0.00765	1	1	Wikipathways
Steroid hormone biosynthesis - homo sapiens (human)	0.0103	1	2	KEGG
Acyl chain remodeling of CL	0.0115	1	1	Reactome
Acyl chain remodeling of PC	0.0115	1	1	Reactome
Hydrolysis of LPC	0.0115	1	1	Reactome
Phosphatidylcholine catabolism	0.0115	1	1	Wikipathways
RXR and RAR heterodimerization with other nuclear receptor	0.0115	1	1	PID
COPI-independent golgi-to-ER retrograde traffic	0.0133	1	1	Reactome
phospho-PLA2 pathway	0.0133	1	1	Reactome
Vitamins A and D - action mechanisms	0.0133	1	1	Wikipathways
Vitamin D (calciferol) metabolism	0.0152	1	1	Reactome
Golgi-to-ER retrograde transport	0.0171	1	1	Reactome
Metabolism of steroids	0.0171	1	2	Reactome
Vitamin D metabolism	0.0171	1	1	Wikipathways
GPCR downstream signalling	0.0185	1	2	Reactome
1_25-dihydroxyvitamin D_3_ biosynthesis	0.019	1	1	HumanCyc
Choline metabolism in cancer - homo sapiens (human)	0.019	1	1	KEGG
HDL remodeling	0.019	1	1	Reactome
Plasma lipoprotein remodeling	0.019	1	1	Reactome
Metabolism of lipids	0.0197	1	3	Reactome
Ca-dependent events	0.0209	1	1	Reactome
Intra-Golgi and retrograde Golgi-to-ER traffic	0.0209	1	1	Reactome
Vitamins	0.0209	1	1	Reactome
Vitamin D3 (cholecalciferol) metabolism	0.0228	1	1	EHMN
Plasma lipoprotein assembly_ remodeling_ and clearance	0.0247	1	1	Reactome
Vitamin D-sensitive calcium signaling in depression	0.0247	1	1	Wikipathways
Signaling by GPCR	0.0264	1	2	Reactome
RAS and bradykinin pathways in COVID-19	0.0266	1	1	Wikipathways
G-protein mediated events	0.0303	1	1	Reactome
PLC beta mediated events	0.0303	1	1	Reactome
16p11.2 proximal deletion syndrome	0.0322	1	1	Wikipathways
Drug Induction of Bile Acid Pathway	0.034	1	1	Wikipathways
Membrane Trafficking	0.0378	1	1	Reactome
Opioid Signaling	0.0378	1	1	Reactome
Recycling of bile acids and salts	0.0378	1	1	Reactome
Linoleate metabolism	0.0396	1	1	EHMN
Vitamin A and carotenoid metabolism	0.0396	1	1	Wikipathways
ADORA2B mediated anti-inflammatory cytokines production	0.0415	1	1	Reactome
G alpha (s) signalling events	0.047	1	1	Reactome
Glycerophospholipid metabolism - Homo sapiens (human)	0.047	1	1	KEGG

## Discussion

The stratification of asthma in groups with the idea of finding the underlying mechanisms has been pursued since a long time ago. Particularly, finding the endotypes of uncontrolled severe asthma is crucial, as the therapeutic options for these patients are limited and often do not work (38). Particularly, in non-allergic asthma, there are limited available options, as this phenotype has been understudied (39, 40). Here, we aimed to elucidate the contribution of allergy to the uncontrolled severe allergic phenotype.

Clinical differences in allergic and non-allergic non-Th2 asthma have been already described in previous publications (3, 10). In our research, we found that UCNA patients were older, had a later onset age, and lower levels of total IgE than UCA patients, which is consistent with these previous publications. Nonetheless, this should be taken into account, as these could act as confounding factors given the complexity of this asthma model. Moreover, AERD was clearly associated with non-allergic asthma, as has been already described (3, 13). It is known that NSAIDs intolerance is not related to an increase in atopy (41). Despite the risk of AERD patients to develop a systemic reaction and the worsening of symptoms after NSAIDs intake, there is a lack of studies on the underlying mechanisms of this syndrome. Interestingly, it has been described that AERD was associated with an increase in eicosanoids compared to aspirin-tolerant asthma (42). However, the allergic status of non-AERD patients was not described; thus, it remains unclear if AERD and allergy activate similar pathways in asthma, and further studies investigating this should be pursued.

Regarding the metabolomic analysis, we found a distinctive metabolic profile between UCA and UCNA patients, which was characterized by a substantial increase in a set of LPCs, including LPC 18:2 and LPC 20:4, two of the most abundant LPCs in plasma (43). These LPCs are related to the PLA2 pathway, which, together with a reduction in palmitoleylcarnitine, point toward the arachidonic acid pathway as critical to understand the contribution of allergy to these settings.

Changes in LPCs have already been described in asthma and allergy (44–47) and extensively associated with inflammatory response (48). Increases in LPC 18:2 and 20:4 have been observed in the active group compared with the placebo treatment in grass-pollen allergic patients after 2 years of sublingual immunotherapy (49), pointing toward an induction of inflammation.

It has been described that mast cells, alveolar macrophages and neutrophils can secrete sPLA2, a process that is triggered by allergen challenge (50). Moreover, during inflammation, sPLA2 is associated with high-density lipoproteins (HDL), and can significantly alter HDL composition, which, in inflammation, are enriched in LPCs, including LPC 18:2. It has been demonstrated that these LPCs can act as mediators and impair platelet aggregation (51). There is increasing evidence of platelets playing a role in asthma (52) and allergy (53, 54).

LPC 20:4 has been associated with increased release of inflammatory cytokines in endothelial cells, triggering M2 or alternative macrophage polarization (55). Furthermore, PLA2 hydrolyses phospholipids and releases LPC and free fatty acids; including arachidonic acid (56–58). Arachidonic acid (AA, C 20:4), which was found increased in its LPC form in UCA, is a precursor of eicosanoids (prostaglandins, lipoxins, leukotrienes), resolvins, and protectins, all of them involved in the regulation of immune response (59). These are known to play a role in asthma, allergy, and other inflammatory pathologies (60, 61).

Interestingly, we found that palmitoleoylcarnitine was decreased in UCA patients. A decrease in carnitines together with an increase in LPCs has been described in severe allergy compared to controls (53).

Overall, our results show that allergic inflammation in uncontrolled asthma leads to a significant alteration of various inflammatory routes, including the AA pathway, along with a dysregulation of LPC mediators, pointing toward these two factors as allergic contributors in asthma.

Steroid biosynthesis was also found increased in UCA patients, which has also been described in allergic asthma compared to healthy controls (47). Steroids have been demonstrated to play a role in the activation of immune system and in the development of asthma (62); thus this might be related with the higher activation shown. Some studies have linked inhaled corticosteroids with a suppression of the adrenal gland (63). However, all our patients were treated with a combination of inhaled corticosteroids and a long-acting bronchodilators; and there were no differences in topical or oral corticosteroid intake among groups. Moreover, there also seemed to be an involvement of bile acids. The role of bile acids in inflammatory diseases is becoming increasingly clearer (64); in particular, there seems to be a connection between respiratory diseases and gastroesophageal reflux; and bile acid increases have been reported previously (45).

Interestingly, deoxycholic acid was found reduced in UCA patients compared to UCNA. We have previously reported a decrease in deoxycholic acid in asthma according to severity (28). This secondary bile acid has been demonstrated to stimulate the production of inflammatory cytokines in an *in vitro* epithelial airway cell culture; and to promote inflammation in animal models either by inducing inflammatory cytokine production (65), activation of the inflammasome (66) and inducing the dysbiosis of gut microbiota (67). Thus, steroid metabolism seems to be differential and a key player in inflammation for non-allergic asthma.

Nontheless, it is important to consider that this is an exploratory study. Future projects including more samples that explore other aspects of asthma are needed. For example, there is increasing evidence that sensitization to Staphylococcus aureus enterotoxins is related with the development of severe asthma and disease exacerbations (68), and with nasal polyps (69). Thus, sensitization to staphylococcal enterotoxins might play a role in non-allergic asthma pathogenesis, and its analysis could be helpful in understanding the influence of asthma in this disease.

## Conclusions

Overall, we found that allergic inflammation elicits a differential inflammatory phenotype in severe uncontrolled asthma patients. This inflammation is related to the arachidonic acid and the PLA2 pathways and is marked by a distinctive metabolomic profile in both groups. Moreover, this study highlights the need of further studies to better elucidate the underlying pathways in non-allergic asthma, as novel therapeutic targets unrelated to type 2 inflammation are needed for a better treatment in these patients.

## Data availability statement

The datasets presented in this study can be found in online repositories. The names of the repository/repositories and accession number(s) can be found below: https://www.ebi.ac.uk/metabolights/, MTBLS1133.

## Ethics statement

The studies involving human participants were reviewed and approved by Comité de ética de la Investigación Hospital Universitario de Gran Canaria Check dot after Dr. Negrin Hospital Barranco de la Ballena s/n. Hospital Universitario de Gran Canaria Dr. Negrín, Edificio de Investigación, Planta principal. 35019 Las Palmas de Gran Canaria (study code: 160009). The patients/participants provided their written informed consent to participate in this study.

## Author contributions

ME was the PI and together with DB and AV designed the study and supervised the research. TC, AA, and HG recruited the patients and obtained the samples. MD, DO, JR-C, AV, and CB performed the metabolomic analysis and data treatment. MD performed analysis of the results together with DO. All authors contributed to the writing of the manuscript and have given approval to the final version of the manuscript.

## Funding

This work was supported by ISCIII (PI19/00044 and PI18/01467), cofounded by FEDER Investing in your future for the thematic network and cooperative research centers ARADyAL (RD16/0006/0015) and RICORS Red de Enfermedades Inflamatorias (REI) (RD21/0002/0008), the Ministry of Science and innovation in Spain (PCi2018-092930) co-funded by the European program ERA HDHL – Nutrition & the Epigenome, Project Dietary Intervention in Food Allergy: Microbiome, Epigenetic and Metabolomic Interactions DIFAMEM and Fundación Mutua Madrileña (AP177712021). MD and JR-C were supported by FPI-CEU predoctoral fellowships and DO was funded by a postdoctoral research fellowship from the European program ERA HDHL – Nutrition & the Epigenome, Project Dietary Intervention in Food Allergy: Microbiome, Epigenetic and Metabolomic Interactions DIFAMEM.

## Conflict of interest

The authors declare that the research was conducted in the absence of any commercial or financial relationships that could be construed as a potential conflict of interest.

## Publisher's note

All claims expressed in this article are solely those of the authors and do not necessarily represent those of their affiliated organizations, or those of the publisher, the editors and the reviewers. Any product that may be evaluated in this article, or claim that may be made by its manufacturer, is not guaranteed or endorsed by the publisher.
